# *FGFR3-TACC3* fusion as a potential primary resistance mechanism to EGFR-TKI in lung adenocarcinoma harboring co-driven mutations: a case report

**DOI:** 10.3389/fonc.2026.1780493

**Published:** 2026-03-12

**Authors:** Xiuwen Wang, Liwen Qiu, Jizhen Liang, Fei Liu, Xiaoxue Ma, Pan Yang

**Affiliations:** 1 Department of Oncology, Guangzhou Red Cross Hospital, Jinan University, Guangzhou, China; 2 Hangzhou Astrocyte Technology Co., Ltd., Hangzhou, China

**Keywords:** co-mutations, *EGFR*-TKI, *FGFR3-TACC3* fusion, NSCLC, resistance

## Abstract

Oncogenic driver mutations were once considered mutually exclusive in non-small cell lung cancer (NSCLC), and the optimal management for these patients with co-mutations of driver genes remains controversial. We report a 66-year-old never-smoking female patient with *EGFR* exon 19 deletion (19del) metastatic NSCLC. Progression occurred after around seven months of first-line treatment with osimertinib. After the progression, the molecular testing revealed *CCDC6-RET* fusion in a liver metastasis, two novel *RET* fusions (*IL6ST-RET* and *SLC41A3-RET*), and an *ALK* fusion with a mutation allele frequency of 0.19% in circulating tumor DNA (ctDNA), including the known *EGFR* 19del. Pralsetinib was added to osimertinib, resulting in a response lasting 4 months. Molecular detection of both liver and ctDNA revealed the presence of *ALK* fusions, while *EGFR* 19del still existed, but *RET* fusions disappeared. After one month with alectinib only, osimertinib was added due to the progression, resulting in another response of more than two months. Upon progression with quadruple alterations (*EGFR* 19del, *EGFR* C797S, *MET* amplification, and *RET* fusions), cabozantinib-gefitinib combination was initiated, leading to rapid deterioration. Interestingly, an *FGFR3-TACC*3 fusion was detected at baseline before EGFR-TKI initiation and persisted throughout the patient’s treatment course. The patient died about 18 months after the initial diagnosis of metastatic NSCLC. This case demonstrates that iterative molecular profiling in metastatic NSCLC identifies actionable alterations to optimize clinical management. At the same time, comprehensive genomic testing remains essential for therapeutic decision-making, with ctDNA analysis complementing tissue-based approaches. Notably, the *FGFR3-TACC3* fusion may represent a novel resistance mechanism contributing to the limited efficacy of EGFR-TKI.

## Introduction

Non-small cell lung carcinoma (NSCLC) is the predominant form of lung cancer, with lung adenocarcinoma (LUAD, approximately 80%-85% of cases) and lung squamous cell carcinoma representing its most common histological subtypes (1). Advancements in targeted therapy and next-generation sequencing (NGS) technology have transformed NSCLC management into precision medicine-based strategies.

Guidelines recommend serial genomic profiling of tumor tissue in metastatic NSCLC to inform evolving treatment strategies throughout the disease course (2, 3). Circulating tumor DNA (ctDNA) released into the bloodstream enables comprehensive genetic profiling of all cancerous lesions through liquid biopsy (4, 5). ctDNA overcomes the spatial and temporal limitations inherent to single-site tissues, particularly when tissue sampling is impractical, and facilitates minimally invasive procedures (6). Its clinical utility extends to dynamic monitoring of tumor genomic evolution and therapeutic response, and early detection of resistance mechanisms (7–9). Furthermore, quantitative ctDNA dynamics correlate with treatment efficacy and show emerging value in defining molecular residual disease (10). Longitudinal ctDNA profiling helped decipher the complex resistance landscape and guide therapy, underscoring its important role in precision oncology.

In NSCLC, oncogenic drivers such as *EGFR*, *BRAF*, *MET* exon14, *KRAS* mutations, and *ALK*, *ROS1*, *RET*, and *NTRK* rearrangements have been identified, and the presence of driver mutations is considered to be mutually exclusive (11). However, the expanding application of broad panels has significantly increased molecular screening for driver mutations at diagnosis. A study found that 5% of driver alterations in LUAD involved double or multiple mutations (12). Consequently, the detection of co-occurring driver alterations in lung cancer is increasingly recognized. Detection of targetable driver mutations typically prompts the initiation of matched targeted therapies. However, the clinical implications of co-occurring genomic alterations remain incompletely characterized, necessitating judicious treatment selection in this molecular context.

Oncogenic fibroblast growth factor receptors (FGFRs) alterations, including fusions, have been implicated in the pathogenesis and progression of various malignancies (13–15). Furthermore, the co-occurrence of *FGFR3-TACC3* fusions with *EGFR* mutations has been reported, wherein the *FGFR3* fusion has been identified as a mechanism of acquired resistance to EGFR tyrosine kinase inhibitors (TKIs) in lung cancer patients (16). Here, we report a case of advanced LUAD who had multiple metastases at the initial diagnosis with co-driven mutations, including *EGFR* mutation and *FGFR3* fusion, and was treated with targeted therapy, and developed various driver variations during the course of her disease. In this context, we use the term “primary resistance” to describe a clinical outcome pre-determined by a pre-existing molecular co-alteration. It manifests as an abbreviated response duration to first-line targeted therapy in the absence of canonical secondary resistance mutations of the targeted pathway at initial progression. The persistent *FGFR3* fusion is hypothesized to represent the molecular substrate of this primary resistance in our case, enabling rapid tumor adaptation.

## Case presentation

**Figure 1:**
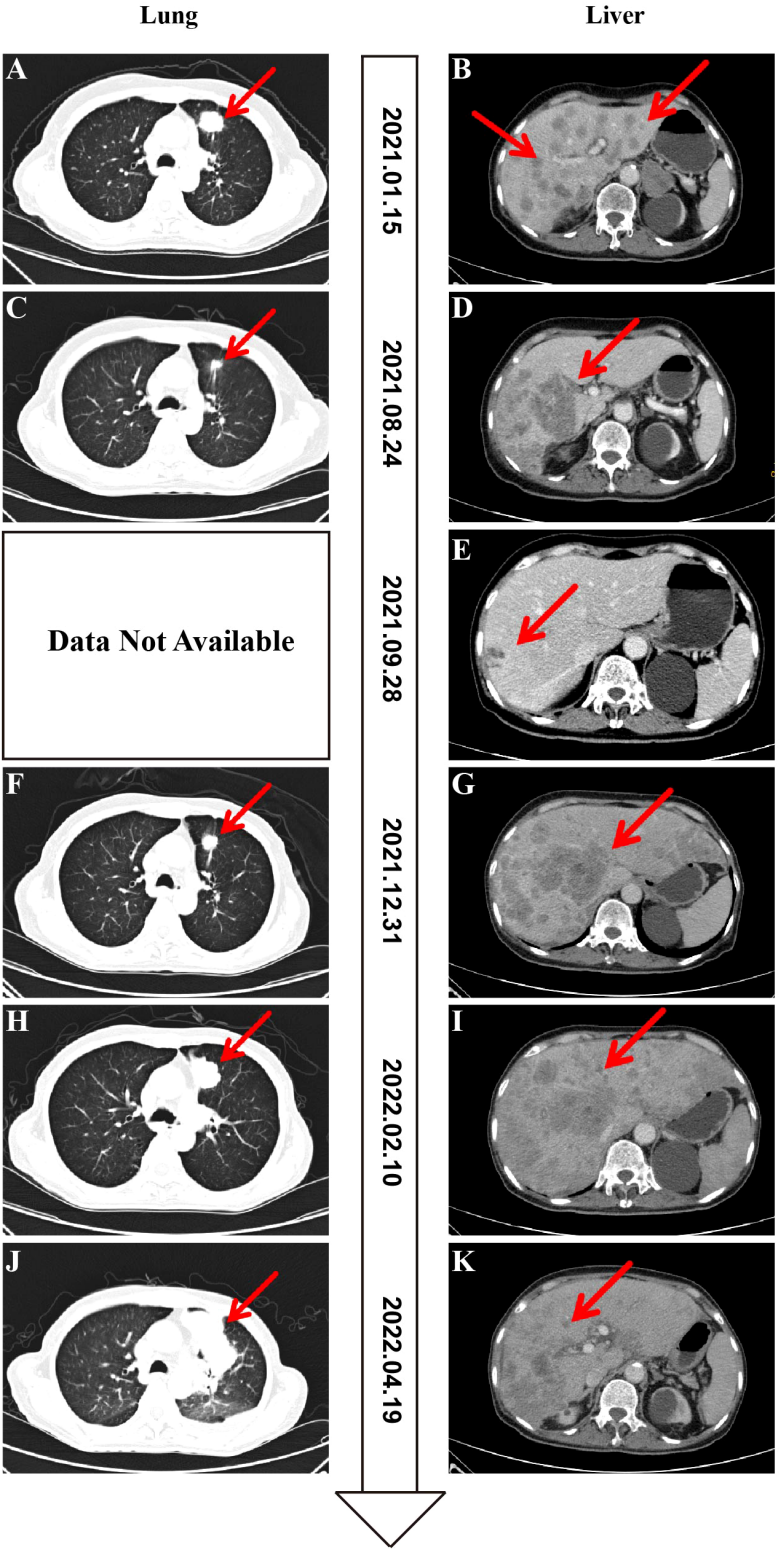
Computed tomography (CT) of this patient at diagnosis and follow-up. **(A, B)** Baseline CT at diagnosis of the lung **(A)** and liver **(B)**. **(C, D)** CT after 7.5 months of osimertinib therapy with decreased size of the left lung **(C)** and progressive disease of the liver **(D)**. **(E)** CT after around 1 month of osimertinib plus pralsetinib therapy with decreased size and number of liver metastases. **(F, G)** CT after 4 months of osimertinib plus pralsetinib therapy with progressive disease of the left lung **(F)** and liver **(G)**. **(H, I)** CT after 1 month of alectinib therapy with progressive disease of the left lung **(H)** and stable disease of the liver **(I)**. **(J, K)** CT after 3 months of alectinib plus osimertinib therapy with progressive disease of the left lung **(J)** and stable disease of the liver **(K)**.

**Figure 2:**
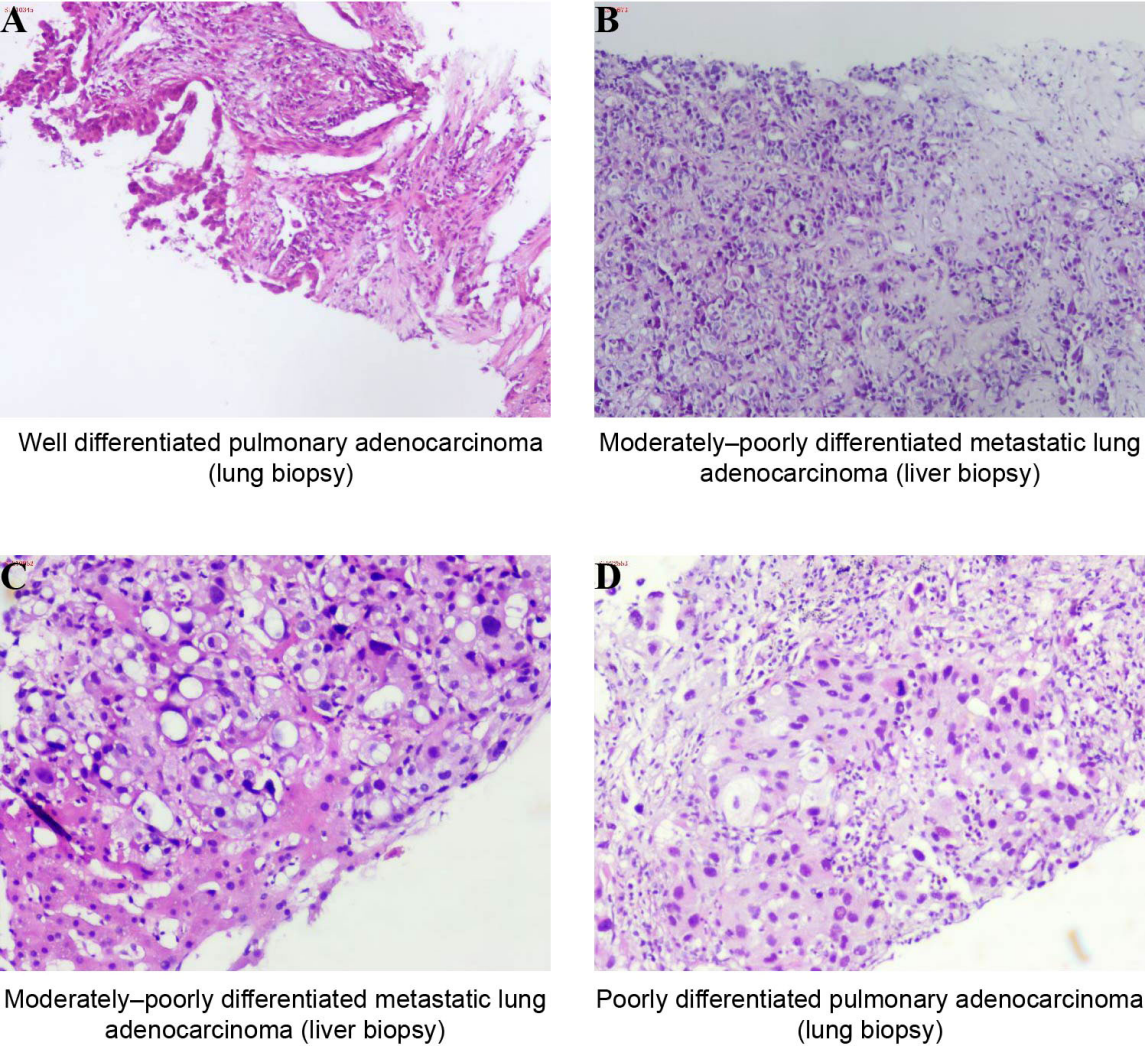
Pathological morphological features of the lung and liver biopsy specimen from the patient. **(A)** The biopsy result of the lung lesion at baseline indicates adenocarcinoma showing well differentiation. **(B, C)** The biopsy results of the liver lesion in August 2021 **(B)** and December 2022 **(C)**, respectively, exhibit features of moderately-poorly differentiated carcinoma. **(D)** The biopsy result of the lung lesion in April 2022 indicates poorly differentiated pulmonary adenocarcinoma. Hematoxylin and eosin (HE) staining, ×100.

On January 15, 2021, a 66-year-old, never-smoking woman presented to our hospital with persistent discomfort in the right upper abdomen for five months and pain for one week. Computed tomography (CT) imaging demonstrated multiple masses in the bilateral lungs (**Figure 1A**), liver (**Figure 1B**), and bones. Further brain magnetic resonance imaging (MRI) indicated the presence of multiple masses in the brain. A transthoracic needle biopsy was performed, and the samples from the left lung mass were confirmed to be adenocarcinoma, with pathological results showing well differentiation (**Figure 2A**). Immunohistochemistry (IHC) staining of the tumor biopsy was positive for Ki-67 (30%), Napsin-A, thyroid transcription factor-1 (TTF-1), cytokeratin (CK), and CK20, but negative for P40 and P53. Based on these findings, the patient was diagnosed at stage cT4N2M1c (stage IVB). PD-L1 immunohistochemistry testing of the same biopsy specimen demonstrated high expression (tumor proportion score, TPS = 60%).

UMI-based sequencing was performed on the NovaSeq 6000 platform, using 2 × 151 bp paired-end reads and a target raw depth of 7,000x for biopsy tissue and more than 30,000x for plasma, with two DNA-based panels that included over 100 cancer-related genes. NGS analysis revealed an *EGFR* exon 19 deletion (19del) with a mutation allele frequency (MAF) of 31.25% in the tissue and 39.94% in ctDNA, alongside a *TP53* mutation (20.16% in tissue; 33.89% in ctDNA). Additional alterations detected exclusively in ctDNA included an *FGFR3*-*TACC3* fusion (17.41%) and *CDKN2A* copy number loss (copy number, CN = 1.1). Given that the *EGFR* 19del was the predominant and canonical oncogenic driver, the patient was initiated on first-line osimertinib (80mg once daily) per standard guidelines.

After achieving an initial partial response (PR), the patient exhibited a PFS of over 6 months. In August 2021, the patient presented for follow-up evaluation due to recurrent epigastric pain and persistent hiccups. MRI demonstrated a marked regression in the bilateral occipital lobe lesions, with virtual resolution of the other intracranial metastatic foci. CT revealed a slight reduction in the primary left upper lobe and resolution of most pulmonary metastases (**Figure 1C**). While partial disappearance of hepatic metastases was observed, new coalescent lesions had formed conglomerate masses in the liver (**Figure 1D**), indicating progressive disease (PD). The histopathological results of liver biopsy, exhibiting features of moderately to poorly differentiated carcinoma, were consistent with the metastasis of lung adenocarcinoma (**Figure 2B**). Genetic testing results showed that hepatic metastatic tissue detected *EGFR* 19del (49.93%), *CCDC6*-*RET* fusion (2.96%), *FGFR3*-*TACC3* fusion (20.32%), *ADCY2*-*TERT* fusion (8.60%; previously reported exclusively in melanoma), and *TP53* mutation (46.47%), while ctDNA profiling revealed *EGFR* 19del (51.13%), *ALK* fusion (0.19%), three distinct *RET* fusions (*IL6ST*-*RET* [1.88%], *CCDC6*-*RET* [1.16%], *SLC41A3*-*RET* [0.34%]), and *FGFR3-TACC3* fusion (30.14%), demonstrating significant spatial heterogeneity with emerging resistance-associated alterations. Given the persistence of the primary *EGFR* 19del driver, a combination targeted strategy was pursued. In the second line, combination therapy with osimertinib (80mg once daily) and a reduced dose of pralsetinib (200mg once daily, RET-TKI) was initiated on 3 September 2021. The pralsetinib dose was halved prophylactically to mitigate the increased risk of toxicities from the osimertinib combination. A follow-up abdominal CT performed on September 28, 2021, demonstrated a significant reduction in both the number and size of hepatic metastatic lesions compared with prior imaging (**Figure 1E**).

Three-month follow-up CT of the thorax and abdomen revealed interval progression of metastatic disease, characterized by a significant increase in both number and size of pulmonary and hepatic lesions, with coalescent liver metastases forming new conglomerate masses (**Figures 1F, G**). Liver biopsy on 31 December 2021, moderately to poorly differentiated, confirmed metastatic from lung adenocarcinoma (**Figure 2C**), with NGS analysis revealing persistent *EGFR* 19del (47.09%) alongside emerging biallelic *ALK* rearrangements (intergenic-*ALK* 16.27% and *EML4-ALK* 0.33%), two functionally distinct *FGFR3-TACC3* fusion isoforms (F17-T14, 24.81% and F17-T10, 0.09%), consistently detected *ADCY2-TERT* fusion (10.73%), and *TP53* mutation (39.78%), while notably lacking acquired *EGFR* resistance mutations (T790M/C797S). Given that the liver was the dominant site of progression, alongside the emergence of *ALK* fusions and clearance of the previously targeted *RET* fusions, alongside cost considerations, the therapeutic strategy was adjusted to alectinib (600mg twice daily) on 8 January 2022 in third-line to prioritize targeting this new resistance pathway, resulting in clinical improvement that allowed discharge with continuation of outpatient *ALK* inhibition. However, one month later, CT on 10 February 2022 demonstrated PD at the primary left lung lesion (**Figure 1H**), with solid enhancement and reduction in multiple liver metastases (**Figure 1I**). Recognizing that the original *EGFR* 19del persisted, osimertinib (80mg once daily) was later re-introduced alongside alectinib upon subsequent progression, aiming to achieve dual-pathway control and address the ongoing clonal evolution of the tumor. This time, a PR with a clinical benefit could be observed for two months with the combination therapy.

**Figure 3:**
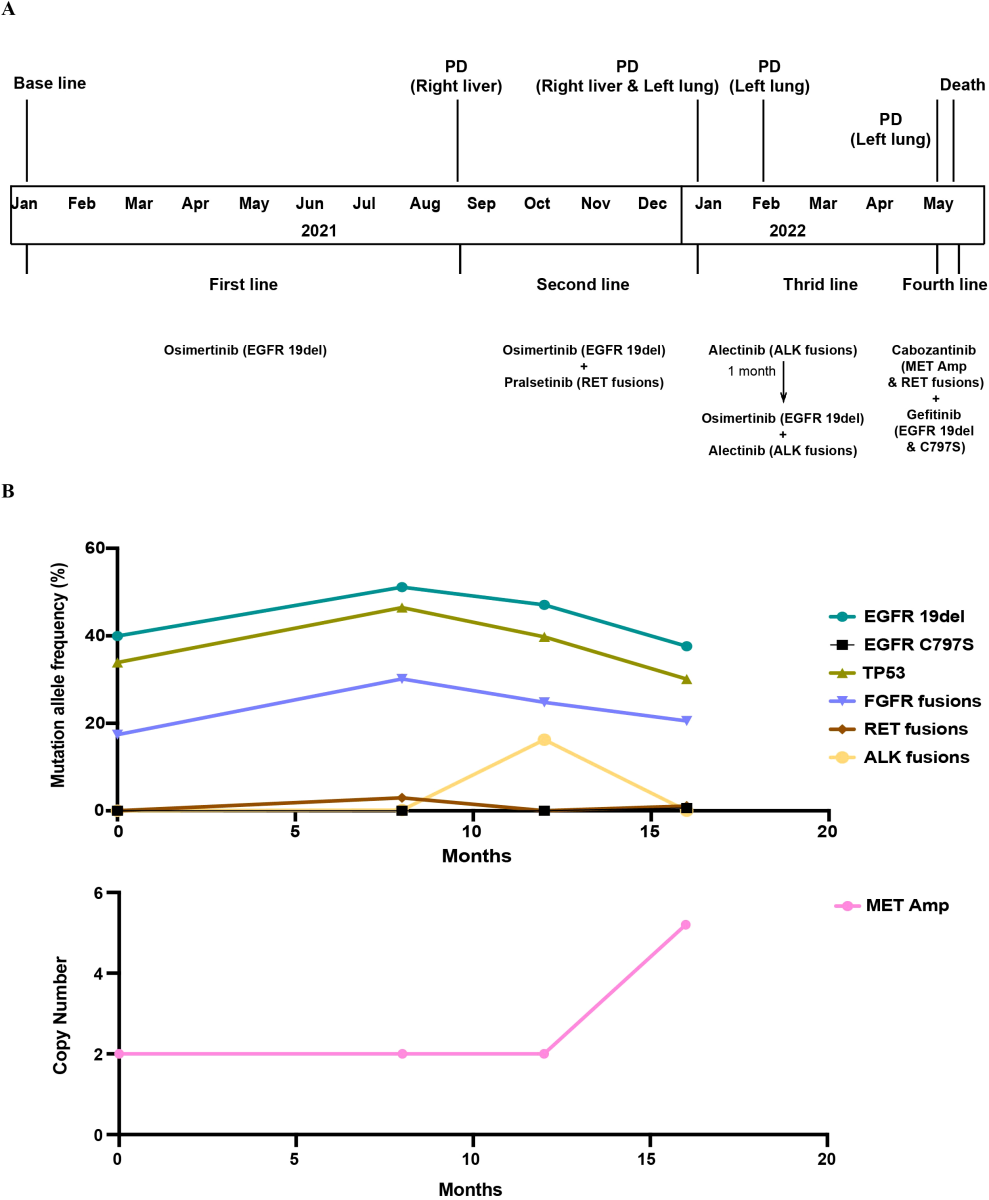
Longitudinal treatment course and dynamic changes of major genomic alterations. **(A)** Treatment timeline. **(B)** Dynamic changes of mutant allele frequency (MAF, %) and copy number (CN) of key genomic alterations over the disease course. PD, progressive disease.

In April 2022, the patient was hospitalized due to persistent fever with worsening cough. CT revealed interval enlargement of the primary left upper lobe lung (**Figure 1J**) and progressive enlargement of the left hilar lymph nodes. As for the liver, the number of multiple metastases had decreased compared to before, but some lesions in the right lobe of the liver still fuse into masses (**Figure 1K**). Pathological examination of a left lung biopsy confirmed transformation to poorly differentiated pulmonary adenocarcinoma (**Figure 2D**), indicating heightened malignant potential. Molecular profiling of the left lung biopsy revealed *EGFR* 19del (30.25%), *MET* amplification (CN = 5.2), *TERT* promoter mutation (3.46%), and *TP53* mutation (16.23%). In comparison, concurrent plasma ctDNA analysis demonstrated *EGFR* 19del (37.62%), biallelic *EGFR* T790M-independent resistance mutation (C797S, 0.65%), *RET* fusions (*IL6ST*-*RET* 1.08% and *CCDC6*-*RET* 0.53%), *FGFR3*-*TACC3* fusion isoforms (F17-T14, 20.55% and F17-T10, 0.22%), *TP53* mutation (30.10%), and *CDKN2A* copy number loss (CN = 1.3). The persistent *EGFR* 19del was now accompanied by a newly acquired *EGFR* C797S mutation, a mutation that confers resistance to osimertinib but can re-sensitize tumors to gefitinib. This was compounded by *MET* amplification in the biopsied lesion and the re-emergence of *RET* fusions in ctDNA. With no single approved TKI capable of addressing all these alterations, the combination of gefitinib (to target the *EGFR* 19del and C797S) and cabozantinib (to concomitantly inhibit *MET* and *RET*) was empirically initiated in a “divide-and-conquer” strategy. In the fourth line, the combination regimen of gefitinib and cabozantinib was initiated on 2 May 2022 and terminated on 23 May 2022 following the onset of refractory diarrhea. Unfortunately, rapid clinical deterioration culminated in death 6 days post-treatment discontinuation, over 16 months after the initial diagnosis of metastatic NSCLC. The timeline of the patient’s treatment history and molecular findings is represented in **Figure 3**.

## Discussion

In this report, we describe a patient with a complex molecular landscape of metastatic LUAD harboring concurrent multiple targetable drivers (*EGFR* mutations, *RET/ALK/FGFR3* fusions, and *MET* amplification). To our knowledge, there is little information available on the therapeutic efficacy of multiple TKIs for these patients. Optimizing targeted therapy selection remains clinically complex in this context, particularly due to the absence of evidence-based guidelines defining the therapeutic hierarchy among different TKIs. Serial molecular testing at PD represents a critical strategy for guiding subsequent therapy in advanced NSCLC patients receiving targeted agents, as evidenced by our case.

Tissue biopsy is considered the gold standard for genomic testing, yet it is limited by temporal and spatial heterogeneity. Plasma cell-free DNA (cfDNA), particularly ctDNA, has emerged as a minimally invasive adjunct that can capture broader molecular heterogeneity, identify actionable alterations in patients with multi-metastatic disease, and enable real-time monitoring of tumor evolution and resistance (17–19). This case unveils appreciable spatiotemporal heterogeneity, with the integration of tissue and ctDNA analyses providing a more comprehensive profile of the patient’s tumor molecular alterations. For example, before the fourth line, lung metastases were dominated by *MET* amplification, while ctDNA revealed *RET* fusions and an *EGFR* C797S mutation, which was undetectable in tissue biopsies. This may suggest that the patient had developed resistance to osimertinib and required a change in treatment strategy. However, the liver-derived *RET* fusions detected at second-line progression were likely overrepresented in ctDNA due to high tumor shedding from this site, illustrating the potential for ctDNA profiling to disproportionately reflect clones from highly vascular metastases. Similarly, the discordant MAFs of the *FGFR3-TACC3* fusion between tissue (20.32%) and ctDNA (30.14%) further reveal organ-specific adaptive selection under targeted therapy pressure. Notably, ctDNA provided an 18.4-week lead time over radiographic imaging in detecting the emergent *ALK* fusion (at 0.19% MAF), demonstrating its unique sensitivity for early detection of clinically relevant subclones. Conversely, the very low MAF of this fusion also raises the possibility that its tissue of origin may have been a low-shedding or anatomically constrained lesion, and that without ultra-sensitive ctDNA profiling, this resistance driver might have been missed entirely. The same assay that enables early detection of certain clones in ctDNA may underrepresent others due to variable shedding across metastatic sites. The discordant MAFs of the *FGFR3-TACC3* fusion between tissue and ctDNA (20.32% vs 30.14%) further exemplify this phenomenon, with the overrepresentation in plasma likely reflecting dominant shedding from high-burden liver metastases. Nevertheless, discrepancies between tissue and ctDNA are not uncommon, arising from tumor heterogeneity, differential shedding, and inherent technical differences between platforms. In this complex, multi-lesion case with concurrent driver alterations, we prioritized therapeutic decisions based on the dominant progressing lesion and mutations with high MAF, limited each regimen to a maximum of two-drug combinations to mitigate cumulative toxicity and financial burden, and maintained close radiographic and molecular surveillance of untreated clones.

Targeted therapy remains the preferred treatment for patients with co-mutations of driver genes; however, determining the appropriate TKI still poses a challenge. In patients harboring multiple-driver variants, genetic testing can provide clues regarding which oncogenic aberration predominates. Relative levels of phosphorylated *EGFR* (p-*EGFR*) may predict the efficacy of targeted therapies for *EGFR*-mutant tumors (20). Likewise, gene abundance may serve as a predictive factor for targeted therapy response (21, 22). At second-line, concurrent tissue and ctDNA analysis revealed high-abundance *EGFR* 19del and *RET* fusions. Targeting these alterations, osimertinib (for *EGFR*) and pralsetinib (for *RET*) were administered. Although an *ALK* fusion was detected in ctDNA, its negligible MAF (0.19%) precluded targeted intervention. And the patient achieved a PFS of 4 months, with subsequent molecular re-evaluation demonstrating clearance of the *RET* fusion. This case highlights the necessity of hierarchical therapeutic targeting based on quantitative molecular profiling, where high-abundance oncogenic drivers warrant immediate intervention while low-frequency alterations require vigilant monitoring for potential clonal evolution during treatment. In addition to targeting variants with high MAF, the expression of dominant clones in progressing lesions is also a critical consideration in treatment. For instance, following disease progression in liver metastases with low MAF of *RET* fusions, the use of RET-TKI in the second line leads to significant shrinkage of the liver metastases.

TKIs have become the preferred first-line treatment option for *EGFR*-mutation advanced NSCLC patients. However, most patients still develop drug resistance. Further cracking the resistance mechanism, including co-mutations, is the key to improving the efficacy of targeted therapy. Studies have shown that patients with *EGFR-TP53* co-mutations have a shorter response rate and PFS when treated with *EGFR*-TKI (23, 24). At present, the mechanism by which *TP53-EGFR* co-mutation leads to *EGFR*-TKI resistance in patients with advanced NSCLC remains unclear, but it may be related to changes in cell function and signaling pathways. High tumor burden with liver-dominant disease may have further compromised the treatment efficacy of EFGR-TKI (25). Serial biopsies revealed progressive loss of glandular differentiation (from moderately to poorly differentiated adenocarcinoma), suggesting that loss of glandular differentiation could facilitate more rapid adaptive resistance (26). Within this complex landscape, the persistently detected *FGFR3-TACC3* fusion, present at baseline, sustained throughout treatment, and unaccompanied by secondary *EGFR* mutations at initial progression, represents an interesting case-specific hypothesis. FGFRs are a family of transmembrane receptor tyrosine kinases (RTKs) and mediate critical functions in a spectrum of essential physiological processes. As key regulators, FGFRs orchestrate diverse cellular activities, including cell survival, proliferation, migration, differentiation, and metabolism. Oncogenic *FGFR* alterations, including fusion, are implicated in the pathogenesis and progression of multiple malignancies (27). A large-scale genomic analysis identified *FGFR* aberrations (including gene amplifications, mutations, and rearrangements) in 7.1% of solid tumors, with approximately 13% of these alterations occurring in lung cancer (28).

Fusion events are observed in lung cancer, albeit usually at low frequencies, but may drive acquired/intrinsic resistance to EGFR-TKIs. In a LUAD study with a total incidence of *FGFR3-TACC3* fusion of 0.5%, *FGFR3-TACC3* fusion led to interleukin-3-independent growth of Ba/F3 cells, and the cells were resistant to the *EGFR* inhibitor gefitinib (29). In a large-scale clinical NSCLC cohort, five cases of *FGFR3-TACC3* fusions were identified, all containing the intact kinase domain of *FGFR3* and the coiled-coil domain of *TACC3*. Notably, these fusions emerged after *EGFR*-TKI therapy: one following erlotinib, one after afatinib, one post-osimertinib, and one after ASP8273 treatment (16). Although acquired *FGFR3-TACC3* fusions post-*EGFR* TKI are rare, they demonstrate that this rearrangement represents a potential bypass resistance mechanism in *EGFR*-mutant NSCLC, capable of circumventing *EGFR* blockade by all generations of *EGFR*-TKIs (16, 30). Additionally, it has been demonstrated that *FGFR3-TACC3* fusion proteins act as naturally occurring drivers of tumor resistance by functionally substituting for EGFR/ERK signaling (31). In the case described herein, the *FGFR3-TACC3* fusion was detected at baseline before TKI initiation and persisted throughout the patient’s treatment course, suggesting that it may have intrinsically driven primary resistance. FGFR, ALK, RET, and MET belong to the RTK family, and their activation converges on common downstream signaling pathways (**Supplementary Figure 1**). When targeted drugs block one pathway, cancer cells can reactivate these shared downstream pathways by upregulating FGFR signaling. Studies indicate that FGFR signaling is associated with resistance to TKIs targeting EGFR, ALK, and MET in lung cancer (32). Similar bypass mechanisms may also operate in *RET* fusions, a common *RTK* fusion. Current research evidence on bypass resistance primarily focuses on FGFR3 overexpression or overall activation of the FGFR pathway. However, the *FGFR3* fusion directly leads to constitutive activation of the FGFR pathway and theoretically possesses the potential to drive bypass activation. Consequently, during targeted therapy, the emergence of *FGFR3* fusion may necessitate considering combination strategies that inhibit both FGFR and other driver alterations to overcome bypass-mediated resistance. Given that FGFR-TKIs were not approved for lung cancer during the treatment period of our case, the therapeutic impact of *FGFR* inhibition on outcomes remains undetermined. While no single factor can be definitively causally implicated, we propose that this fusion, in concert with the aforementioned adverse clinical and genomic features, likely contributed to the early treatment failure observed.

Furthermore, the detection of novel fusion partners may hold particular scientific significance. *RET* fusions are the most commonly reported RTK fusions that mediate acquired resistance to *EGFR*-TKIs, and *CCDC6-RET* is the most common fusion. Previous studies demonstrated that *RET* fusions were more likely associated with NSCLC patients treated with third-generation *EGFR*-TKIs, mediated secondary resistance to third-generation *EGFR*-TKIs, and might be associated with poor prognosis (33, 34). The *IL6ST-RET* and *SLC41A3-RET* fusions, previously unreported in lung cancer, have not been directly reported as mechanisms of *EGFR*-TKI resistance, but cannot be ruled out as a possible “bypass activation” mechanism (30). Although initially reported in melanoma (35), the *ADCY2-TERT* fusion may indirectly drive TKI resistance in lung cancer through multifactorial mechanisms, despite lacking direct evidence as a resistance driver. Potential pathways include cAMP/PKA signaling activation, telomere maintenance dysregulation, transcriptional reprogramming, tumor microenvironment modulation, and epigenetic alterations. Future integration of *TERT* fusions into NGS large panels will expand real-world datasets to elucidate their role in resistance mechanisms.

Despite sequential TKI targeting of dominant driver variants at each therapeutic stage, this patient developed drug resistance after each line of treatment. So, it is imperative to explore alternative therapeutic strategies to improve survival. Most approved targeted agents are designed against a single or a limited number of driver alterations. When tumors harbor more than one actionable mutation, multi-drug combinations become necessary, inevitably increasing toxicity, regimen complexity, and cost. Moreover, clonal heterogeneity and bypass pathway activation frequently render single-target inhibition ineffective. Therefore, in patients with multiple actionable drivers, “broad-spectrum” therapeutic strategies merit consideration. The IMpower150 trial evaluated first-line atezolizumab plus bevacizumab, carboplatin, and paclitaxel (ABCP) in advanced non-squamous NSCLC (36). Among *EGFR*-mutant patients who had previously received *EGFR*-TKI, ABCP conferred a better median OS than BCP alone (HR = 0.74), indicating that the quadruplet regimen may be an effective later-line option after *EGFR*-TKI failure. In the Phase II ALTER-L038 study, among advanced *EGFR*-mutant NSCLCs who had progressed on prior EGFR-TKI therapy, the chemo-free combination of benmelstobart plus anlotinib achieved a median OS of 28.9 months (37). The FLAURA2 trial compared osimertinib in combination with platinum-based chemotherapy to osimertinib monotherapy as first-line treatment for patients with advanced NSCLC harboring *EGFR*-sensitizing mutations. Previously reported data from the Chinese subgroup demonstrated a median PFS of over 33 months (HR = 0.58). Newly released results show that the combination therapy group achieved a median OS of 47.5 months (HR = 0.77) (38). In *EGFR*-mutant non-squamous NSCLC following EGFR-TKI treatment failure, previous phase III immunotherapy studies have not demonstrated an OS benefit. The HARMONi-A study randomly assigned patients to receive either ivonescimab (target PD-1 and VEGF-A) plus chemotherapy or chemotherapy alone (39). The most recent OS analysis demonstrated a statistically significant improvement with ivonescimab-chemotherapy (HR = 0.74). Similarly, the ORIENT-31 study demonstrated that patients treated with sintilimab+bevacizumab+pemetrexed+platinum significantly improved mPFS. And the triplet-ICI regimen achieved a mOS of 21.1 months; after adjustment for crossover, the OS hazard ratio was 0.798, indicating clinically meaningful efficacy (40). It is noteworthy that our case exhibited high PD-L1 expression (TPS = 60%) at baseline, yet immunotherapy was not utilized in the first-line treatment. This decision was aligned with contemporary clinical guidelines (e.g., NCCN) and evidence. For patients with treatment-naïve, *EGFR*-mutant metastatic NSCLC, first-line EGFR-TKI therapy is the established standard of care, regardless of PD-L1 status, due to its demonstrated superior efficacy and PFS benefit over immunotherapy. Furthermore, a well-documented heightened risk of severe immune-related adverse events, notably pneumonitis, in *EGFR*-mutant populations treated with immune checkpoint inhibitors warranted caution (41). The subsequent emergence of a complex landscape of co-occurring driver alterations (*RET*, *ALK*) further complicated the predictability of immunotherapy response, supporting the strategy of prioritizing sequential targeted therapies based on iterative molecular profiling. However, later-phase clinical studies have subsequently established a role for combination immunotherapy (with chemotherapy and/or angiogenesis inhibitors) in the management of *EGFR*-mutant NSCLC following resistance to targeted therapies. Thus, incorporating broad-spectrum treatments, such as immunotherapy and chemotherapy, could potentially delay tumor progression. However, determining the optimal timing for broad-spectrum antitumor interventions in patients with driver gene positivity remains challenging. Notably, these combinatorial strategies offer renewed hope for NSCLCs with multiple driver alterations, yet their true benefit awaits validation in larger, prospective datasets.

## Conclusion

In summary, we document a rare and molecularly intricate case of multisite metastatic NSCLC, enriching the current understanding of LUAD harboring concurrent driver alterations. Clinical decision-making for NSCLC patients harboring concurrent actionable drivers faces multidimensional challenges, and exploring potential combinations or sequential therapy strategies is necessary. The persistent presence of *FGFR3-TACC* fusion suggests its potential role in compromising long-term response to EGFR-TKI. For patients experiencing rapid and widespread progression, promptly targeting resistance mechanisms such as *FGFR3* fusions or introducing broad-spectrum antitumor therapy at a judicious time could be a strategy to delay resistance. Moreover, our results demonstrate that comprehensive genomic profiling is essential for therapeutic decision-making in patients with advanced NSCLC, and ctDNA analysis serves as a robust complement to tissue-based genomic profiling.

## Data Availability

The original contributions presented in the study are included in the article/**Supplementary Material**. Further inquiries can be directed to the corresponding author.

## References

[1] Zhou YJ Zheng W Zeng QH Ye Y Wang C Fang C . Targeted exome sequencing identifies mutational landscape in a cohort of 1500 Chinese patients with non-small cell lung carcinoma (NSCLC). Hum Genomics. (2021) 15:21. doi: 10.1186/s40246-021-00320-9, PMID: 33845897 PMC8042687

[2] Hendriks LE Kerr KM Menis J Mok TS Nestle U Passaro A . Oncogene-addicted metastatic non-small-cell lung cancer: ESMO Clinical Practice Guideline for diagnosis, treatment and follow-up. Ann Oncol. (2023) 34:339–57. doi: 10.1016/j.annonc.2022.12.009, PMID: 36872130

[3] Hanna NH Robinson AG Temin S Baker S Jr. Brahmer JR Ellis PM . Therapy for stage IV non-small-cell lung cancer with driver alterations: ASCO and OH (CCO) joint guideline update. J Clin Oncol. (2021) 39:1040–91. doi: 10.1200/JCO.20.03570, PMID: 33591844

[4] de Abreu AR Wyninckx A Vandamme T Op de Beeck K Van Camp G Peeters M . Circulating Tumor DNA detection in cancer: a comprehensive overview of current detection methods and prospects. Oncologist. (2025) 30:oyaf204. doi: 10.1093/oncolo/oyaf204, PMID: 40680231 PMC12450317

[5] Li S Noor ZS Zeng W Stackpole ML Ni X Zhou Y . Sensitive detection of tumor mutations from blood and its application to immunotherapy prognosis. Nat Commun. (2021) 12:4172. doi: 10.1038/s41467-021-24457-2, PMID: 34234141 PMC8263778

[6] Akabane M Imaoka Y Kawashima J Pawlik TM . Advancing precision medicine in hepatocellular carcinoma: current challenges and future directions in liquid biopsy, immune microenvironment, single nucleotide polymorphisms, and conversion therapy. Hepat Oncol. (2025) 12:2493457. doi: 10.1080/20450923.2025.2493457, PMID: 40260687 PMC12026093

[7] Xie LJ Fu LL Liu SC Bai CS Xu BC He XW . Adaptive therapy for perioperative non-small cell lung cancer: strategies guided by dynamic minimal residual disease adjustment. Transl Oncol. (2026) 64:102660. doi: 10.1016/j.tranon.2025.102660, PMID: 41496417 PMC12813118

[8] Busser B Lupo J Sancey L Mouret S Faure P Plumas J . Plasma circulating tumor DNA levels for the monitoring of melanoma patients: landscape of available technologies and clinical applications. BioMed Res Int. (2017) 2017:5986129. doi: 10.1155/2017/5986129, PMID: 28484715 PMC5397613

[9] Jha P Mishra R Joshi A Sharma N Shah M Babu G . Circulating tumor DNA profiling for non-invasive genomic analysis in Indian lung cancer patients: A real-world experience. J Liq Biopsy. (2025) 8:100300. doi: 10.1016/j.jlb.2025.100300, PMID: 40503459 PMC12158504

[10] Abbosh C Hodgson D Doherty GJ Gale D Black JRM Horn L . Implementing circulating tumor DNA as a prognostic biomarker in resectable non-small cell lung cancer. Trends Cancer. (2024) 10:643–54. doi: 10.1016/j.trecan.2024.04.004, PMID: 38839544

[11] Kosaka T Yatabe Y Endoh H Kuwano H Takahashi T Mitsudomi T . Mutations of the epidermal growth factor receptor gene in lung cancer: biological and clinical implications. Cancer Res. (2004) 64:8919–23. doi: 10.1158/0008-5472.CAN-04-2818, PMID: 15604253

[12] Kris MG Johnson BE Kwiatkowski DJ Iafrate AJ Wistuba II Aronson SL . Identification of driver mutations in tumor specimens from 1,000 patients with lung adenocarcinoma: The NCI’s Lung Cancer Mutation Consortium (LCMC). J Clin Oncol. (2011) 29:CRA7506–CRA. doi: 10.1200/jco.2011.29.15_suppl.cra7506, PMID: 41735675

[13] Gu Z Gao S Zhang F Wang Z Ma W Davis RE . Protein arginine methyltransferase 5 is essential for growth of lung cancer cells. Biochem J. (2012) 446:235–41. doi: 10.1042/BJ20120768, PMID: 22708516 PMC3865921

[14] Long A Yamamiya I Valentine M Machnes Z Hangai N Anderson B . A phase I drug-drug interaction study to assess the effect of futibatinib on P-gp and BCRP substrates and of P-gp inhibition on the pharmacokinetics of futibatinib. Clin Transl Sci. (2024) 17:e70012. doi: 10.1111/cts.70012, PMID: 39258521 PMC11388056

[15] Gelbrich N Miebach L Berner J Freund E Saadati F Schmidt A . Medical gas plasma augments bladder cancer cell toxicity in preclinical models and patient-derived tumor tissues. J Adv Res. (2023) 47:209–23. doi: 10.1016/j.jare.2022.07.012, PMID: 35931323 PMC10173201

[16] Ou SI Horn L Cruz M Vafai D Lovly CM Spradlin A . Emergence of FGFR3-TACC3 fusions as a potential by-pass resistance mechanism to EGFR tyrosine kinase inhibitors in EGFR mutated NSCLC patients. Lung Cancer. (2017) 111:61–4. doi: 10.1016/j.lungcan.2017.07.006, PMID: 28838400 PMC10203818

[17] Maia MC Salgia M Pal SK . Harnessing cell-free DNA: plasma circulating tumour DNA for liquid biopsy in genitourinary cancers. Nat Rev Urol. (2020) 17:271–91. doi: 10.1038/s41585-020-0297-9, PMID: 32203306

[18] Lee JS Cho EH Kim B Hong J Kim YG Kim Y . Clinical practice guideline for blood-based circulating tumor DNA assays. Ann Lab Med. (2024) 44:195–209. doi: 10.3343/alm.2023.0389, PMID: 38221747 PMC10813828

[19] Iams WT Mackay M Ben-Shachar R Drews J Manghnani K Hockenberry AJ . Concurrent tissue and circulating tumor DNA molecular profiling to detect guideline-based targeted mutations in a multicancer cohort. JAMA Netw Open. (2024) 7:e2351700. doi: 10.1001/jamanetworkopen.2023.51700, PMID: 38252441 PMC10804266

[20] Yang JJ Zhang XC Su J Xu CR Zhou Q Tian HX . Lung cancers with concomitant EGFR mutations and ALK rearrangements: diverse responses to EGFR-TKI and crizotinib in relation to diverse receptors phosphorylation. Clin Cancer Res. (2014) 20:1383–92. doi: 10.1158/1078-0432.CCR-13-0699, PMID: 24443522

[21] Zhou Q Zhang XC Chen ZH Yin XL Yang JJ Xu CR . Relative abundance of EGFR mutations predicts benefit from gefitinib treatment for advanced non-small-cell lung cancer. J Clin Oncol. (2011) 29:3316–21. doi: 10.1200/JCO.2010.33.3757, PMID: 21788562

[22] Ariyasu R Nishikawa S Uchibori K Oh-Hara T Yoshizawa T Dotsu Y . High ratio of T790M to EGFR activating mutations correlate with the osimertinib response in non-small-cell lung cancer. Lung Cancer. (2018) 117:1–6. doi: 10.1016/j.lungcan.2017.12.018, PMID: 29496249

[23] Wang Y Liu H Yu N Xiang X . Concordance of abundance for mutational EGFR and co-mutational TP53 with efficacy of EGFR-TKI treatment in metastatic patients with non-small-cell lung cancer. Curr Oncol. (2023) 30:8464–76. doi: 10.3390/curroncol30090616, PMID: 37754531 PMC10528559

[24] Takaoka H Terai H Nakamura K Mizuno T Kawano R Emoto K . Clinical application of in-house comprehensive genomic profiling for thoracic cancer: insights from a Japanese hospital. Cancer Sci. (2025) 116:2819–30. doi: 10.1111/cas.70168, PMID: 40757605 PMC12485672

[25] Iso H Yomota M Shirakura Y Yoshinaga T Kawai S Narita K . Clinical impact of osimertinib dose reduction in the first-line setting on EGFR mutation-positive non-small cell lung cancer: A retrospective monocentric study. Onco Targets Ther. (2025) 18:379–87. doi: 10.2147/OTT.S494112, PMID: 40124926 PMC11930247

[26] Zhao J Xu W Zhou F Zhang X Zhou M Miao D . Navigating the landscape of EGFR TKI resistance in EGFR-mutant NSCLC - mechanisms and evolving treatment approaches. Nat Rev Clin Oncol. (2026) 23:63–83. doi: 10.1038/s41571-025-01085-z, PMID: 41219394

[27] Touat M Ileana E Postel-Vinay S Andre F Soria JC . Targeting FGFR signaling in cancer. Clin Cancer Res. (2015) 21:2684–94. doi: 10.1158/1078-0432.CCR-14-2329, PMID: 26078430

[28] Helsten T Elkin S Arthur E Tomson BN Carter J Kurzrock R . The FGFR landscape in cancer: analysis of 4,853 tumors by next-generation sequencing. Clin Cancer Res. (2016) 22:259–67. doi: 10.1158/1078-0432.CCR-14-3212, PMID: 26373574

[29] Capelletti M Dodge ME Ercan D Hammerman PS Park SI Kim J . Identification of recurrent FGFR3-TACC3 fusion oncogenes from lung adenocarcinoma. Clin Cancer Res. (2014) 20:6551–8. doi: 10.1158/1078-0432.CCR-14-1337, PMID: 25294908

[30] He J Huang Z Han L Gong Y Xie C . Mechanisms and management of 3rd−generation EGFR−TKI resistance in advanced non−small cell lung cancer (Review). Int J Oncol. (2021) 59. doi: 10.3892/ijo.2021.5270, PMID: 34558640 PMC8562388

[31] Daly C Castanaro C Zhang W Zhang Q Wei Y Ni M . FGFR3-TACC3 fusion proteins act as naturally occurring drivers of tumor resistance by functionally substituting for EGFR/ERK signaling. Oncogene. (2017) 36:471–81. 10.1038/onc.2016.216 PMC5290037 27345413

[32] Liu X Mei W Yao Y Zeng C . Current insights and barriers in FGFR-mediated signaling in lung cancer. Eur J Med Chem. (2025) 296:117897. doi: 10.1016/j.ejmech.2025.117897, PMID: 40554984

[33] Wang C Zhang Z Sun Y Wang S Wu M Ou Q . RET fusions as primary oncogenic drivers and secondary acquired resistance to EGFR tyrosine kinase inhibitors in patients with non-small-cell lung cancer. J Transl Med. (2022) 20:390. doi: 10.1186/s12967-022-03593-3, PMID: 36059009 PMC9441062

[34] Zhu VW Klempner SJ Ou SI . Receptor tyrosine kinase fusions as an actionable resistance mechanism to EGFR TKIs in EGFR-mutant non-small-cell lung cancer. Trends Cancer. (2019) 5:677–92. doi: 10.1016/j.trecan.2019.09.008, PMID: 31735287

[35] Liang WS Hendricks W Kiefer J Schmidt J Sekar S Carpten J . Integrated genomic analyses reveal frequent TERT aberrations in acral melanoma. Genome Res. (2017) 27:524–32. doi: 10.1101/gr.213348.116, PMID: 28373299 PMC5378171

[36] Socinski MA Nishio M Jotte RM Cappuzzo F Orlandi F Stroyakovskiy D . IMpower150 final overall survival analyses for atezolizumab plus bevacizumab and chemotherapy in first-line metastatic nonsquamous NSCLC. J Thorac Oncol. (2021) 16:1909–24. doi: 10.1016/j.jtho.2021.07.009, PMID: 34311108

[37] Shi M Chen P Cui B Yao Y Wang J Zhou T . Benmelstobart plus anlotinib in patients with EGFR-positive advanced NSCLC after failure of EGFR TKIs therapy: a phase I/II study. Signal Transduct Target Ther. (2024) 9:283. doi: 10.1038/s41392-024-01982-2, PMID: 39389963 PMC11467201

[38] Planchard D Jänne PA Kobayashi K Yang JCH Liu Y Valdiviezo N . PL02.06 first-line osimertinib + Chemotherapy versus osimertinib monotherapy in EGFRm advanced NSCLC: FLAURA2 final overall survival. J Thorac Oncol. (2025) 20:S1. doi: 10.1016/j.jtho.2025.09.015, PMID: 41800429

[39] Zhang L Fang W Zhao Y Luo Y Yang R Huang Y . 1348 Final overall survival analysis of HARMONi-A study comparing ivonescimab plus chemotherapy to chemotherapy alone in patients with EGFR+ NSCLC progressed on EGFR-TKI treatment. J ImmunoTherapy Cancer. (2025) 13. doi: 10.1136/jitc-2025-SITC2025.1348, PMID: 41802225

[40] Lu S Wu L Jian H Cheng Y Wang Q Fang J . Sintilimab plus chemotherapy for patients with EGFR-mutated non-squamous non-small-cell lung cancer with disease progression after EGFR tyrosine-kinase inhibitor therapy (ORIENT-31): second interim analysis from a double-blind, randomised, placebo-controlled, phase 3 trial. Lancet Respir Med. (2023) 11:624–36. doi: 10.1016/S2213-2600(23)00135-2, PMID: 37156249

[41] Bruno D Dowlati A . Immunotherapy in EGFR mutant non-small cell lung cancer: when, who and how? Transl Lung Cancer Res. (2019) 8:710–4. 10.21037/tlcr.2019.06.02 PMC6835106 31737508

