# Supplementary material for: FGFR3-TACC3 fusion as a potential primary resistance mechanism to EGFR-TKI in lung adenocarcinoma harboring co-driven mutations: a case report
Source: Front Oncol. 2026 Mar 12;16:1780493. doi: 10.3389/fonc.2026.1780493 (PMC13017310; doi:10.3389/fonc.2026.1780493)
Supplement: Supplementary file 1 [file Table1.docx]

Supplementary Material

## Supplementary Figures


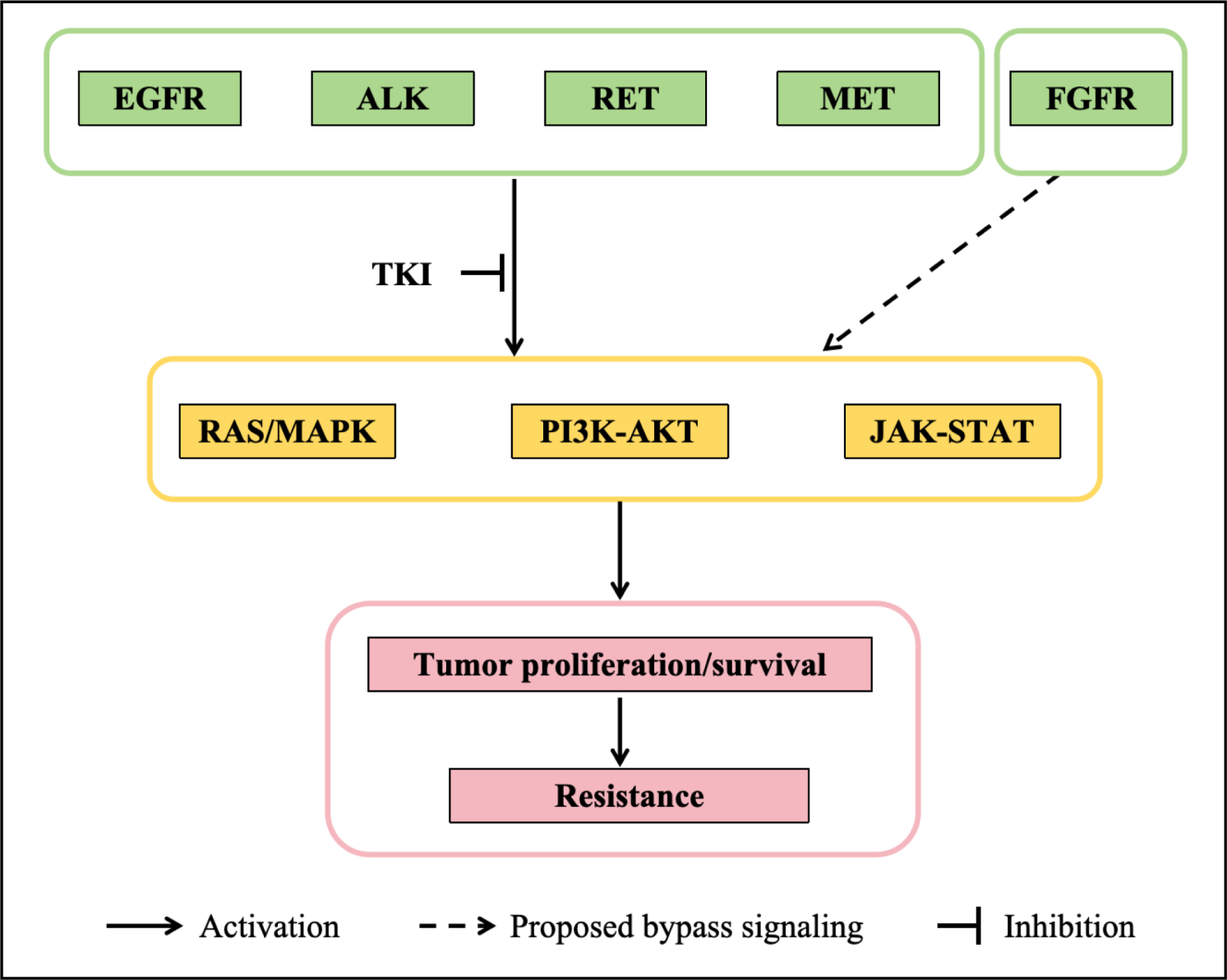


**Supplementary Figure 1.** **Schematic model of convergent RTK signaling and FGFR3‑TACC3‑mediated bypass resistance.** Solid arrows indicate established downstream activation cascades. T‑bar lines represent inhibition by corresponding targeted therapies. Dashed arrows denote the hypothesized bypass signaling driven by the constitutively active FGFR3‑TACC3 fusion,.
